# Genetic Characterization of Chinese fir from Six Provinces in Southern China and Construction of a Core Collection

**DOI:** 10.1038/s41598-017-13219-0

**Published:** 2017-10-23

**Authors:** Hongjing Duan, Sen Cao, Huiquan Zheng, Dehuo Hu, Jun Lin, Binbin Cui, Huazhong Lin, Ruiyang Hu, Bo Wu, Yuhan Sun, Yun Li

**Affiliations:** 1 Beijing Advanced Innovation Center for Tree Breeding by Molecular Design. National Engineering Laboratory for Tree Breeding, Key Laboratory of Genetics and Breeding in Forest Trees and Ornamental Plants, Ministry of Education, College of Biological Sciences and Technology, Beijing Forestry University, 100083 Beijing, People’s Republic of China; 2 Guangdong Provincial Key Laboratory of Bio-control for the Forest Disease and Pest, Guangdong Academy of Forestry, 510520 Guangzhou, People’s Republic of China; 3 The ex situ gene bank of Longshan State Forest Farm, 512221 Guangzhou, Guangdong Province People’s Republic of China; 4 Department of Biochemistry, Baoding University, 071000 Baoding, Hebei Province People’s Republic of China; 5 Fujian Jiangle State-owned Forestry Farm, Fujian, 353300 China

## Abstract

Large *ex situ* germplasm collections of plants generally contain significant diversity. A set of 700 well-conserved Chinese fir (*Cunninghamia lanceolata* (Lamb.) Hook) clones from six provinces in southern China in the *ex situ* gene bank of Longshan State Forest, was analyzed using 21 simple sequence repeat markers, with the aim of assessing the genetic diversity of these germplasm resources. Genetic analysis revealed extensive genetic variation among the accessions, with an average of 8.31 alleles per locus and a mean Shannon index of 1.331. Excluding loci with null alleles, we obtained a low level of genetic differentiation among provinces, consistent with the interpopulation genetic variation (1%). Three clusters were identified by STRUCTURE, which did not match the individuals’ geographical provenances. Ten traits related to growth and wood properties were quantified in these individuals, and there was substantial variation in all traits across individuals, these provide a potential source of variation for genetic improvement of the Chinese fir. Screening large collections for multiple-trait selective breeding programs is laborious and expensive; a core collection of 300 accessions, representative of the germplasm, was established, based on genotypic and phenotypic data. The identified small, but diverse, collections will be useful for further genome-wide association studies.

## Introduction

Genetic variation is essential for the adaptability of a population and is the basis for the evolutionary potential of a species^1,2^. Many plant species are composed of a large number of individuals distributed across vast areas and thus may be rich in diversity. Understanding their genetic variability is important for efficient selection and maintenance of germplasm collections^3,4^; this is especially true for tree species, which are perennial, woody, contain a large number of individuals, and are usually cross-pollinating^5^. A considered approach to the preservation of plant genetic resources is vital. *Ex situ* germplasm collections are essential for the conservation of plant genetic resources, and they generally involve significant diversity. Appropriate evaluation of *ex situ* collections of trees, including an estimate of the genetic structure and diversity of populations, provides not only an understanding of their genetic relationships^6,7^ and an ability to establish core collections^8,9^ but also important information for association mapping^10,11^.

To assess genetic variability, morphological characteristics are often used, although they can be affected by environmental conditions. As an alternative, molecular markers are more stable and reliable for use in the characterization of germplasm resources and have been used to characterize the genetic variability of various species at the DNA level^12,13^. Among various molecular markers, simple sequence repeat (SSR) markers are the most popular, as they are co-dominant, hypervariable, neutral, and highly informative^14^. Association analysis, which is based on relating genes or loci to traits, is often used to identify relationships between morphological characteristics and molecular markers or candidate genes to improve the efficient management and utilization of plant genetic resources. However, association mapping of large germplasm collections is laborious. To improve conservation and the effective use of genetic resources, core collections^15^ are often used as materials for association analyses^16,17^.

The Chinese fir [*Cunninghamia lanceolata* (Lamb.) Hook], belonging to the *Taxodiaceae* family, which is diploid (2n = 2x = 22)^18^, is the principal indigenous tree species in subtropical southern China. It is an economically valuable conifer with high yield, good wood quality, high pest resistance, and many uses, including as furniture or paper material. The species is monoecious, with a predominance of outcrossing, although self-pollination is also likely^19^. Studies on the genetic modification of Chinese fir have been performed since 1957, including provenance tests, plus-tree selection, clonal tests, and so on; thus, many seed plantations, progeny forests, germplasm banks, and so forth have been built, which have provided many good germplasm resources^20^. In recent years, the area of artificial afforestation of Chinese fir has expanded consistently, and fourth-generation seed orchards have now been established. These provide potential sources of beneficial alleles for Chinese fir breeding and improvement. Resolution of the genetic structure and relatedness of the accessions would allow proper quantitative analysis of the progeny and would also form the basis of molecular marker-assisted selection breeding. Hence, a more comprehensive analysis of genetic diversity and population structure in Chinese fir is essential. Previous studies have focused on characterizing the genetic variation^21–26^ of Chinese fir; however, the number of samples evaluated was relatively small, consisting of fewer than 150 accessions. In 2004, a set of 700 Chinese fir trees from the provinces of Guangxi (GX), Jiangxi (JX), Hunan (HN), Guizhou (GZ), Fujian (FJ) and Guangdong (GD) were conserved in the *ex situ* gene bank of Longshan State Forest Farm, Guangdong Province, China (25°11’N, 113°28’E, 285–296 m above sea level). Understanding the genetic background of these germplasm resources and their appropriate evaluation is important for their effective use. In addition, a core collection is needed to establish a program of molecular marker-assisted selection breeding to improve the efficient use of Chinese fir in the future.

Therefore, in this study, 10 growth- and wood-property traits were measured in 2014, and SSR markers were used to evaluate the genetic variability of this germplasm resource. Then a core collection that can represent the whole collection was built.

## Results

### Genetic Diversity among the Loci

Twenty-one SSR markers were used to evaluate the genetic diversity among 700 clones of the Chinese fir from six different provinces. All of these were neutral SSR markers not affected by natural selection. A total of 181 alleles were identified across the loci, ranging from 3.83 at SSR3 to 18.83 at SSR1 (Table S1). The number of effective alleles per marker ranged from 1.39 to 11.05, with an average of 3.80. The mean value for *I* was 1.331, ranging from 0.588 to 2.601. The mean *H*o was 0.561, which differed from the *H*e value (0.604). Of the 21 primers used to characterize the Chinese fir collection, *H*o was lower than *H*e at 14 loci. Five loci (SSR1, SSR2, SSR6, SSR 11, and SSR21) showed significant deviation from Hardy–Weinberg equilibrium (HWE) due to heterozygote deficiency or null alleles. Possible null alleles were identified in four loci (SSR1, SSR2, SSR11, and SSR21), with null allele frequencies ranging from 0.06 to 0.29. Of these, SSR1, SSR11, and SSR21, which had higher null allele frequencies, were excluded from the following analyses. The mean fixation index (*F*_IS_) was 0.053 (P < 0.05). Low genetic differentiation among the loci was observed based on F-statistics. The PIC value ranged from 0.25 (SSR12) to 0.92 (SSR1), with an average of 0.57, indicating that the loci were reasonably informative (Table S1).

### Population Structure of the Chinese fir Samples

**Figure 1:**
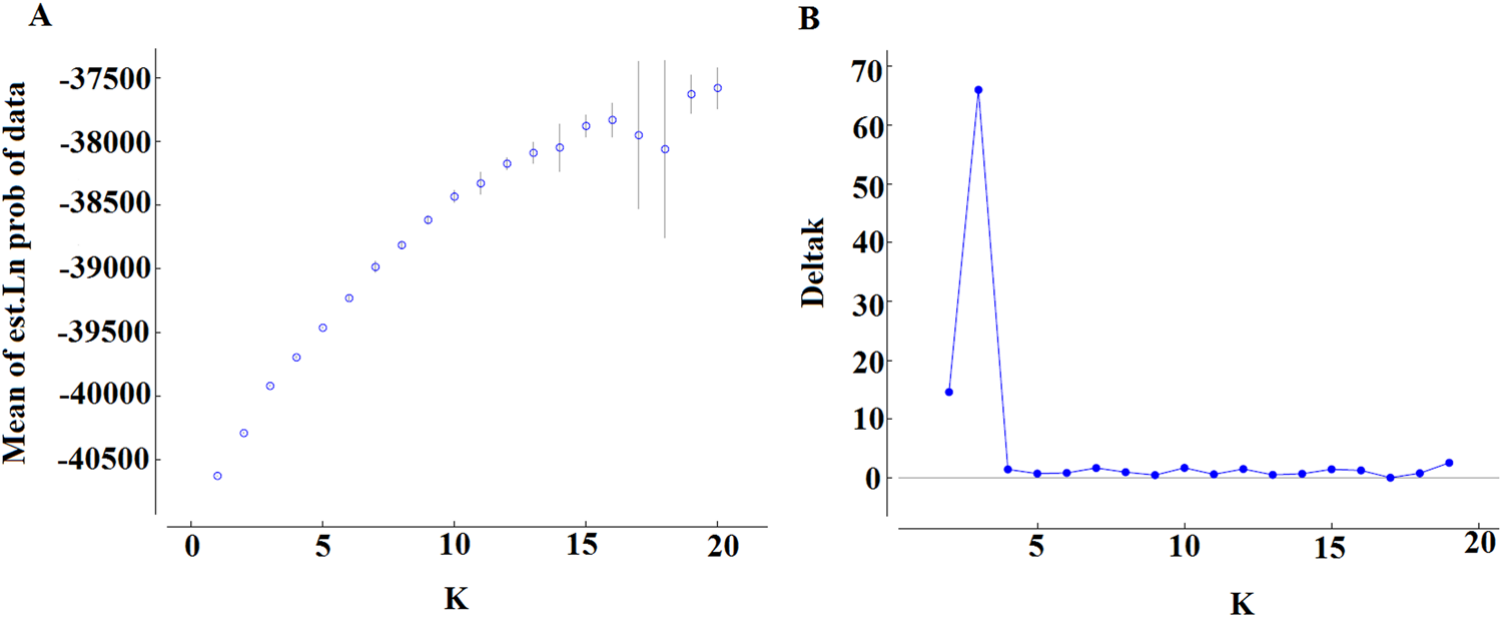
Plot showing Ln P (D) ± SD and ΔK values. (**A**). The mean Ln P (D) was based on 10 repeats for each K value. (**B**). Plot showing ΔK according to K.

**Figure 2:**
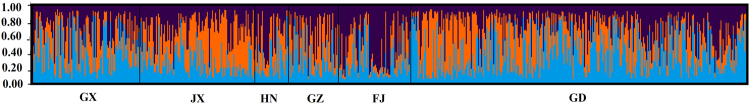
Genetic structure of the 700 Chinese fir individuals. Each individual is represented by a vertical line divided into segments representing the estimated membership proportion in the three genetic clusters inferred with STRUCTURE (GX: Guangxi; JX: Jiangxi; HN: Hunan; GZ: Guizhou; FJ: Fujian; GD: Guangdong).

The analysis of the optimal substructure of the genetic relationship among Chinese fir accessions, excluding loci with null alleles, showed a clear Δ*K* peak at *K* = 3 (Δ*K* = 66.0) (Fig. 1); further evidence regarding K values, which ranged from 1 to 20 using Marverick software, also showed that K = 3 was the most likely value in this study (Figure S1), indicating that all individuals grouped into three major clusters. Among the 10 independent replicate runs with k = 3, the major mode showed exactly identical patterns of individual assignment in each run (Figure S2). Cluster 3 contained fewer individuals (Cluster 3: n = 222) than did Cluster 1 (Cluster 1: n = 237) and Cluster 2 (Cluster 2: n = 241) (Table S2), with average Q values of 0.628, 0.633, and 0.606, respectively. Sixty-one individuals from JX (53.98%) and 18 individuals from GZ (36.73%) belonged to Cluster 1. Over half the individuals from the HN and FJ provinces belonged to Cluster 3, with proportions of 51.52% and 59.15%, respectively. Clusters 1, 2, and 3 included 34.29%, 41.90%, and 23.81% of the individuals from GX and 24.62%, 41.64%, and 33.74% of the individuals from GD, respectively. The average membership coefficient in the entire accession was 0.622. In terms of estimated membership probability (*Q*), only 15.43% individuals showed ancestry values >0.80, and 343 individuals showed ancestry values <0.60 (Table S3); the population structure was weak, and most individuals from the six provinces belonged to a genetic cluster (Fig. 2). The three clusters were dominated by different gene pools but showed inconspicuous genetic differentiation from each other.

The number of alleles and their frequency in the three clusters showed a certain degree of difference (Table S4). The highest allele frequency (0.903) had a size of 216 bp on SSR17 in Cluster 1. The minimum allele frequency that can be measured was 0.002. Among 176 alleles in 18 loci, there were 118 alleles that could be detected in all clusters, accounting for 67.05%. The remaining 58 rare alleles were detected in parts of the clusters.

### Genetic Diversity among Chinese fir Populations

Population genetic parameters, including *N*a, *N*e, *I*, *H*o, and *H*e, were calculated using microsatellite data excluding loci with null alleles to estimate the variation among the samples obtained from six different provinces, as well as the three clusters, at the structure level (Table 1). Among the six provinces, the value of *N*a and the number of private alleles in GD was higher than those in other provinces, which may have been due to the larger sample size^27^. HN contained the fewest individuals, had the lowest *N*a, and lacked any private alleles. The three genetic clusters contained 241, 243, and 216 individuals, respectively, with each cluster having a similar value of *Na* and private alleles. Despite large disparities in sample size among the six provinces, there was little variation in the level of genetic diversity, which corresponded to the level of each cluster. The highest value of *N*e was 4.007, seen in JX, while the lowest value (3.409) was seen in HN. The highest *H*o and *H*e values in FJ were not significantly higher than the lowest values in GX, with differences of 0.035 and 0.023, respectively. The value of *H*o was lower than that of *H*e in all six provinces, but this difference was not significant, which is consistent with the positive fixation index value. Genetic differentiation between any two provinces was calculated for all six provinces using pairwise genetic differentiation values (*F*_ST_) (Table S5). Most of the pairwise tests of differentiation (*F*_ST_) performed between locations were significant (P < 0.01), with an overall *F*_ST_ value of 0.009, suggesting that there was weak differentiation among the six provinces. The values ranged from 0.003 (GD-GX; GD-JX) to 0.016 (HN-FJ). A similar pattern of differentiation among provinces was observed using the standard Nei’s genetic distance estimate (Table S5). Rousset’s genetic distance values [*F*_ST_ /(1– *F*_ST_)] (1997) also indicated that HN is most distant from other provinces, whereas GD-GX and GD-JX were most closely related. Further analysis among the three clusters yielded a mean value for *F*_ST_ of 0.010, while the mean Nei’s genetic distance was 0.034, indicating weak differentiation among them.

**Table 1:** Genetic diversity parameters for all provinces and all populations of the Chinese fir.

Populations	Sample size	*Na*	*Ne*	*I*	*H*_*O*_	*He*	*F*	Private alleles
Guangxi	105	8.619	3.792	1.322	0.548	0.602	0.073	3
Jiangxi	113	8.524	4.007	1.352	0.570	0.602	0.030	6
Hunan	33	6.429	3.409	1.242	0.561	0.590	0.033	0
Guizhou	49	7.619	3.916	1.340	0.550	0.604	0.084	4
Fujian	71	8.143	3.697	1.368	0.583	0.625	0.053	2
Guangdong	329	10.524	3.978	1.353	0.554	0.601	0.053	20
Mean		8.310	3.8	1.331	0.561	0.604	0.054	
Cluster 1	241	9.524	3.756	1.342	0.573	0.604	0.034	16
Cluster 2	243	9.667	3.818	1.320	0.556	0.593	0.042	17
Cluster 3	216	9.524	3.654	1.338	0.546	0.602	0.075	17

*N*a: Number of Different Alleles; *N*e: Number of Effective Alleles; *I*: Shannon’s Information Index; *H*_O_: Observed Heterozygosity; *H*e: Expected Heterozygosity; F: Inbreeding Coefficient.

### Analysis of Molecular Variance (AMOVA)

AMOVA was performed for all Chinese fir accessions of the six different provinces to analyze the distribution of genetic diversity among and within the provinces. The results revealed low variation among the provinces (Table 2). Nearly all the variation (99%) was attributed to differences within provinces. A hierarchical AMOVA of the three genetic clusters using STRUCTURE revealed that 96% of the variance was distributed within the clusters.

**Table 2:** Analysis of molecular variance from microsatellite data excluding loci with null alleles using GenAlEx 6.5.

Source	df	Sum of squares	MS	Est. Var.	%	*P* -value
Variance partition^a^						
Among the provinces	5	156.357	31.271	0.175	1	<0.01
Within a province	694	9571.798	13.792	13.792	99	<0.01
Total	699	9728.154		13.967	100	
Variance partition^b^						
Among the clusters	2	261.454	130.727	0.502	4	<0.01
Within a cluster	697	9466.700	13.582	13.582	96	<0.01
Total	699	9728.154		14.084	100	

^a^The first analysis included all provinces. ^b^The second analysis included three genetic clusters.

### Phenotypic Data Analysis

Ten growth- and wood-property traits were measured in all 700 clones. The growth traits included tree height (H), diameter at breast height (DBH), bark thickness (T), and stem volume (V). The wood-property traits included proportion of heartwood (P), wood basic density (WBD), hygroscopicity (Hy), tracheid length (L), tracheid diameter (D), and the ratio of L to D (L/D). Abundant phenotypic variation was detected among the 700 clones (Table 3). Data from outlier trees were discarded. The coefficient of variation was > 10%, and that of stem volume was highest, at >70.5% and a range from 0.0015 to 0.3235 m^3^. Tracheid length had the lowest coefficient of variation (11.9%). The maximum tree height (15.25 m) was more than 10-fold that of the minimum tree height (1.50 m), and the average height was 7.71 m. Further analyses of the phenotypic variation in the three clusters are shown in Table 4. ANOVA for the three clusters revealed there were no significant differences in most traits, except for P. The components of variance in all measured traits (Table 5) also showed that the environmental variance in each trait was much less than the genetic variance, suggesting that environmental effects were small for these traits, corresponding to the small value of Q_ST_.

**Table 3:** The minimum and maximum values, mean, standard error (SE) and coefficient of phenotypic variation [*CV* (%)] for each phenotypic trait measured in the Chinese fir.

Statistics	H (m)	DBH (cm)	T (cm)	V (m^3^)	P (%)	WBD (g/cm^3^)	Hy (%)	L (µm)	D (µm)	L/D
Minimum	1.5	3.40	2.33	0.0015	6.43	0.2286	130.60	1863.26	25.25	35.68
Maximum	15.25	24.27	9.00	0.3235	45.55	0.5298	438.11	3580.89	70.64	108.20
Mean	7.71	13.23	4.60	0.0737	21.61	0.3151	259.46	2720.09	46.15	62.15
SE	2.16	3.54	0.79	0.0519	6.39	0.0408	39.53	322.73	6.28	10.51
CV (%)	28.02	26.76	17.17	70.47	29.57	12.95	15.24	11.86	13.61	16.91

H: tree height; DBH: diameter at breast height; T: bark thickness; V: stem volume; P: proportion of heartwood; WBD: wood basic density; Hy: hygroscopicity; L: tracheid length; D: tracheid diameter; L/D: the ratio of L to D.

**Table 4:** Differences in 10 traits of Chinese fir.

Population	Mean									
	H	DBH	T	V	P	WBD	Hy	L	D	L/D
Cluster1	7.49 ± 0.08a	12.82 ± 0.13b	4.50 ± 0.03b	0.0684 ± 0.0019b	20.45 ± 0.23b	0.3170 ± 0.0015a	257.07 ± 1.46a	2705.23 ± 12.14a	45.92 ± 0.22a	61.99 ± 0.37a
Cluster2	7.79 ± 0.08a	13.23 ± 0.13ab	4.61 ± 0.03ab	0.0743 ± 0.0020ab	21.71 ± 0.24a	0.3156 ± 0.0017a	259.74 ± 1.57a	2705.14 ± 11.70a	45.80 ± 0.25a	62.42 ± 0.41a
Cluster3	7.84 ± 0.08a	13.64 ± 0.14a	4.69 ± 0.03a	0.0786 ± 0.0019a	22.70 ± 0.24a	0.3127 ± 0.0014a	261.54 ± 14.61a	2751.64 ± 12.30a	46.78 ± 0.24a	61.94 ± 0.40a
MS	7.853	37.204	1.767	0.006	0.026	0.001	0.101	137782.387	55.311	14.023
F – value	1.682	2.985	2.875	2.169	6.547	0.589	0.645	1.322	1.400	0.128
P – value	0.187	0.051	0.057	0.115	0.002	0.555	0.525	0.267	0.247	0.880

H: tree height; DBH: diameter at breast height; T: bark thickness; V: stem volume; P: proportion of heartwood; WBD: wood basic density; Hy: hygroscopicity; L: tracheid length; D: tracheid diameter; L/D: the ratio of L to D.

**Table 5:** Genetic components in traits of Chinese fir.

Variance Components	H	DBH	T	V	P	WBD	Hy	L	D	L/D
V_G_	3.6809	9.3635	0.4691	2.20E-03	0.0016	0.0011	0.1022	95253	34.05	102.10
V_E_	0.0047	0.1248	0.0051	8.76E-06	0.0001	0	0	0	0.00	2.16 E-13
Residual	2.1764	7.1037	0.4052	1.60E-03	0.0084	0.0015	0.1411	46569	19.90	69.64
Q_ST_ %	0.13	1.32	1.08	0.40	5.88	0	0	0	0	0

H: tree height; DBH: diameter at breast height; T: bark thickness; V: stem volume; P: proportion of heartwood; WBD: wood basic density; Hy: hygroscopicity; L: tracheid length; D: tracheid diameter; L/D: the ratio of L to D.

### Development of a Core Collection

**Figure 3:**
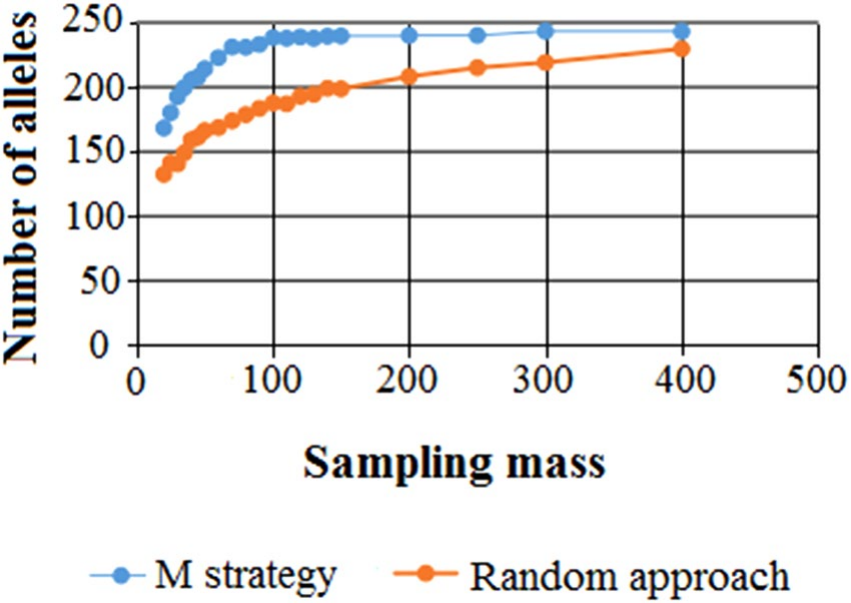
Schematic relationship between the sampling mass and the number of alleles.

To determine the appropriate sampling strategy and optimal core size, different sampling percentages from the whole collection were designed, and this was combined with an M strategy and random sampling method. The average build results for five repeats (Fig. 3) revealed that the number of alleles reserved by the M strategy was always greater than the number reserved by the random sampling method when the core germplasm was constructed using the same sample collection. The latter exhibited inferior efficiency compared to the former, especially for core sets with smaller sample collections, demonstrating a larger allele retention gap. The number of alleles sampled increased rapidly with increased sampling size using the M strategy; however, when the sampling size reached 150 individuals, the curve gradually levelled out, and there was no obvious change in the number of alleles when the sampling quantity increased. Ultimately, the M strategy was considered the preferred strategy for constructing the core selection and, considering that the core germplasm collected will be used for association analysis in the future, a sampling size of 300 was set. In addition, 10 phenotypic traits, including H, DBH, T, V, P, WBD, Hy, L, D and L/D, were used to construct the core germplasm using POWERCORE, which resulted in the identification of 26 individuals exhibiting 100% of the diversity of the whole collection. Finally, a core germplasm of 300 individuals (52 from GX, 34 from JX, 20 from HN, 20 from GZ, 37 from FJ, and 137 from GD) with complete phenotypes were selected based on the results of both methods described above.

### Comparisons of the Whole Collection with the Core Collection

The phenotypic data and genetic diversity parameters were computed for the core collection (Tables 6 and 7). The lowest coincidence rate of the range of the core collection was 78.76% of the whole collection, and the highest rate of variation of the *CV* among the measured traits was only 17.71%. Additionally, t-tests performed for all phenotypic data showed no significant differences between the core collection and the whole collection, except for H (Table 6). All phenotypic data showed a normal distribution. In addition, the genetic parameters *N*a, *N*e, *I*, *H*o, *H*e and PIC were calculated for the core collections, which were all very close to those of the entire collection and also showed no significant differences (Table 7). Among them, the values of *I* and PIC were as high as 96.84% and 99.22%, respectively, of the whole collection, indicating that the core collection was highly representative of the whole collection of 700 accessions.

**Table 6:** Comparison of phenotypic characteristics between the core collection and whole collection.

Trait	xmax-xmin		coincidence rate of range %	*CV*		The rate of variation of the *CV*	mean		mean difference percentage %	mean T-test
	whole collection	core collection		Whole collection	Core collection		whole collection	core collection		
H(m)	13.75	11.42	83.05	30.06	25.79	16.56	7.71	7.94	2.98	0.05
DBH(cm)	20.87	19.02	91.14	29.31	24.9	17.71	13.23	13.55	2.42	0.1
T(cm)	6.67	6.67	100	20.25	17.21	17.66	4.6	4.6	0	0.95
V(m^3^)	0.3220	0.3166	98.32	77.63	68.38	13.53	0.0737	0.0777	5.43	0.2
P(%)	0.3912	0.3722	95.14	29.58	29.02	1.93	21.61	21.64	0.14	0.93
WBD(g.cm^−3^)	0.3012	0.2816	93.49	12.95	13.5	−4.07	0.3151	0.3156	0.16	0.85
Hy(%)	3.0751	2.4167	78.59	15.25	15.98	−4.57	259.46	259.71	0.10	0.92
L(µm)	1717.63	1717.63	100	11.87	11.74	1.11	2720.09	2727.39	0.27	0.69
D(µm)	45.39	41.95	92.42	13.63	13.54	0.66	46.15	46.03	−0.26	0.75
L/D	61.82	60.45	97.78	16.82	17.18	−2.10	62.13	62.54	0.56	0.54

H: tree height; DBH: diameter at breast height; T: bark thickness; V: stem volume; P: proportion of heartwood; WBD: wood basic density; Hy: hygroscopicity; L: tracheid length; D: tracheid diameter; L/D: the ratio of L to D.

**Table 7:** Comparison of molecular diversity between the core collection and whole collection.

	Whole collection	Core collection	T-test
Na	8.310	7.135	0.066
Ne	3.8	3.641	0.097
I	1.331	1.289	0.09
Ho	0.561	0.553	0.262
He	0.604	0.597	0.264
PIC	0.5748	0.5703	0.924

*N*a: Number of Different Alleles; *N*e: Number of Effective Alleles; *I*: Shannon’s Information Index; *H*_O_: Observed Heterozygosity; *H*e: Expected Heterozygosity; F: Inbreeding Coefficient.

## Discussion

Characterization of the germplasm is essential for germplasm conservation and collection activities. A large sample of Chinese fir individuals from six different provinces was collected, grafted, and grown at a single location. We surveyed this germplasm for growth and wood-property traits, which are all important for the economic value of woody stems^28^. The substantial variation identified provides a potential opportunity for genetic improvement of the Chinese fir. In addition, by using neutral SSR markers, extensive genetic variation was detected. Among the 21 SSR loci, most conformed to HWE, and no population had a particularly large number of loci that deviated from HWE. Four loci may have had null alleles, contributing to positive values for the inbreeding coefficient^29^. *F*_IS_ values differed significantly from 0, suggesting that self-pollination may exist, or the Wahlund effect may be present^30^. The mean heterozygosity values *H*o and *H*e were 0.561 and 0.604, respectively, similar to the values in red-colored heartwood genotypes of Chinese fir in GX province (*H*o = 0.562 and *H*e = 0.584)^26^, and determined using the same microsatellite loci despite the sample size of this study being almost five times that of the previous study. Wang *et al*.^31^ also obtained similar heterozygosity values for different numbers of wild and semi-wild apricots, using the same microsatellites. Number of loci, rather than the number of populations, affects the estimate of genetic diversity, consistent with the report by Ferrer *et al*.^32^. The mean values for *Ho* and *He* obtained using SSR markers in this germplasm were higher than those reported for other Chinese firs^24–26^ and other conifers^33–35^, but lower than those in *Abies chensiensis* and *Austrocedrus chilensis* ^36,37^; this suggests that the level of genetic diversity in this germplasm is moderate. Outcrossing and wind-pollination may explain the considerable level of polymorphisms in this species^38–41^. Of the 21 SSR loci used in this study, 14 showed lower values of *H*o than *H*e, indicating a deficiency in heterozygotes at these loci. The same heterozygote deficiency was observed at the population level excluding null alleles. The results are consistent with previous findings in the Chinese fir^26^ using the same 21 SSR markers. In general, natural selection occurring throughout the life cycle of a tree appears to favor heterozygosity^42^, and hybrids show excess heterozygotes^43^. In this tree species, however, heterozygote deficiency may be explained by self-pollination^41^ or by subpopulation structure^30^. The marker data also showed that the genetic distance between some clones was very small, such as between 25 and 26, 516 and 532, 242 and 244, 185 and 32, 122 and 123, 153 and 154, 533 and 535, 625 and 631, 55 and 61, 171 and 172, 10 and 260, 297 and 305, 96 and 253, 289 and 386, 393 and 400, 515 and 544, 24 and 285, 251 and 266, 529 and 541, and so on. Heterozygosity plays an important role in the response to environmental changes^44^, and therefore maintenance of heterozygosity and retention of heterosis are vital, although further analyses such as Mendelian inheritance testing^1^ are needed to verify heterozygote deficiency. Previous analyses of the phenotypic traits of this species identified 98 relatively fast-growing genotypes with relatively high wood basic density^45^, and all of them had distant genetic distances. These genotypes can be chosen to be the parent in cross breeding. In the future, it will be necessary to further expand the Chinese fir breeding base and increase gene communication between the trees. The presence of private alleles in this germplasm may indicate useful rare variants and also provides the opportunity to select useful recombinants in the future.

In this study, a mean of 8.31 alleles per locus from 700 Chinese fir accessions was obtained, a value higher than those in previous reports^24–26^. The high number of alleles identified in this study may be due to the large sample size^1^. The relatively low *F*_ST_ value (0.015) of loci observed indicated a low level of genetic differentiation. Correspondingly, only 1% genetic variation was seen among provinces, as confirmed by AMOVA. These results confirmed those from a previous study showing that outcrossing in woody plants resulted in increased genetic diversity and reduced genetic differentiation among populations^31^. Furthermore, the levels of genetic diversity in each province except HN were similar, although there was a large disparity in sample size. These results indicate that genetic diversity may not be influenced by the number of samples when a large sample size of Chinese fir is available. The *F*_ST_ values between pairwise provinces were significant but low, which indicated that most have mixed ancestry and therefore clustered together. Rousset’s genetic distance values showed that HN was genetically the most distantly related among the provinces, which may have been due to its small sample size, while the most closely related were GD-GX and GD-JX, which may have been due to their geographical proximity or to human activity, such as artificial cultivation.

STRUCTURE identified three non-distinct clusters among the accessions of Chinese fir. The membership coefficient of the whole accession revealed that only 15.43% of individuals had ancestry values >0.80, and nearly half of the individuals had ancestry values <0.60. ANOVA also revealed no significant differences in most traits among the three clusters. Cluster assignment did not match geographical provenances, indicating that the Chinese fir accessions had a mixed ancestry, consistent with the low *F*_ST_ values obtained. That may explain the small phenotypic variation obtained among provinces in a previous study^45^. The low divergence in this resource could be due to several factors. The materials used in this study were all plus trees, including excellent genetic materials, good local-type materials, excellent provenance materials, and hybrid materials, and may have been widely used. This may have resulted in gene flow under past anthropogenic activities, lowering the differentiation among populations. Additionally, the relatively small geographic scale of our survey may have also been a factor. Previous studies have shown that the influence of climate on population structure is stronger than that of the geographical distance influence in *P. tomentosa*, and *P. tomentosa* clones were generally assigned to three different climate regions, whereas most of the geographical proximities were clustered into different groups^1^. Wang *et al*.^31^ also reported that geographic distance was not the principal factor influencing genetic differentiation in the Siberian apricot. The trees investigated in this study were all from similar climate zones, and a sufficient number of individuals may not have been sampled from all local populations, obscuring the genetic structure of Chinese fir, which may have weakened the genetic structure to a certain extent. In addition, outcrossing rate, population size and life-history traits can also have a strong influence on the genetic structure of plant populations^38–40^. A history of genetic drift may also contribute to the phenomenon of non-conspicuous genetic differentiation^26^. Chinese fir is an economically valuable and widely used conifer that is widely distributed in southern China, covering more than 9. 11 × 10^6^ hm^2^. To obtain greater economic benefits, directional selection is used in breeding, and random sampling from small groups may lead to genetic drift that may contribute to low genetic differentiation. Such past anthropogenic activities may have significantly influenced the present population structure and patterns of genetic diversity in Chinese fir. Additionally, Chinese fir is a wind-pollinated tree species with a low level of inbreeding, which can lead to genetic drift. The samples of this germplasm were derived from different provinces; therefore, a comprehensive study of genetic structure should improve our ability to assess the distances over which differentiation can occur in Chinese fir^38,40^. Understanding the genetic structure of Chinese fir could facilitate the selection of trees for breeding to maximize genetic diversity as well as to enhance the potential gain from selection, which may have an impact on the ecological adaptation and evolution of this species in the future^40^.

Association analysis in multiple-trait selective breeding programs is a breeding strategy that can accelerate the breeding process; however, association mapping of large germplasm collections is laborious. At the same time, to avoid using too small of a sample to assess the correlation between phenotypes and genotypes we selected 300 accessions from different genetic backgrounds, representing the maximum variability of 700 Chinese fir accessions, for future association mapping studies. The t-tests performed for most phenotypic data and all genetic parameters showed no significant differences between the core and the whole collections, indicating that the core collection is a good representation of the original germplasm. The core collection developed in this study will be useful for genome-wide association studies in the future to accelerate breeding programs for the Chinese fir.

## Materials and Methods

### Plant Material and DNA Extraction

Chinese fir is widely distributed in southern China, including Guangdong, Fujian, Zhejiang, and 14 other provinces, as well as in Taiwan (Figure S3). In 2004, 700 Chinese fir plus trees, including excellent genetic materials, good local type materials, excellent provenance materials, and hybrid materials, were collected from six groups based on their geographical locations, including Guangxi, Jiangxi, Hunan, Guizhou, Fujian, and Guangdong provinces (Figure S3 and Table S6)^46^. These materials come from excellent provenance and a family gene collection area established in 1983, a Chinese fir-type gene resources collection area constructed in 1989, and an excellent hybrid materials genetic resources collection area of a second-generation seed orchard constructed in 1992, including more than 50 provenances with a broad genetic basis. The number of Chinese fir individuals in each provenance ranged from 1 to 67. The selection criteria of original plus trees considered growth indices and morphological parameters, including volume of wood, height–diameter ratio, crown diameter ratio, percent of bark, disease resistance, stem straightness, the natural level of training, crown vice, collateral thickness degrees, growth vigor, and so on. The dominant comparative method and comprehensive evaluation method were used to choose plus trees, and the distance between any two individuals was more than 50 m. From 2004, scions of these trees were grafted onto 2-year-old Chinese fir rootstocks in the *ex situ* gene bank of Longshan State Forest Farm, Guangdong Province, China (25°11’N, 113°28’E, 285–296 m above sea level), which is located in a subtropical region with a moderate climate throughout the year and ample rainfall^47^. Each clone had at least four ramets of similar size and vigor. Grafted ramets were planted randomly at the site at a spacing of 3 × 3 m. This study was performed in strict accordance with the recommendations in the Guide for Observation and Field Studies^48^. Total genomic DNA was extracted from the mature leaves of each clone using a QIAGEN Plant DNeasy Kit (QIAGEN, Hilden, Germany) in 2014. The quality and concentration of the extracted DNA were measured using a NanoDrop 2000 Spectrophotometer.

### SSR Genotyping

Twenty-one previously identified microsatellite markers^26,49^ were used to genotype the 700 clones. Amplification was performed in a 25-μL reaction volume containing 1.0 μL genomic DNA (~ 100 ng), 1.0 μL forward primer (10 μM), 1.0 μL reverse primer (10 μM), 12.5 μL 2X QIAGEN Taq Plus PCR MasterMix, and 9.5 μL double distilled water. The reaction was performed, as described previously^26^, in a T100™ thermal cycler, with an initial denaturation step at 94 °C for 5 min, followed by 35 cycles at 94 °C for 30 s, 56/58/62 °C (depending on the annealing temperature of the primer used) for 30 s, and 72 °C for 30 s, with a final extension at 72 °C for 10 min. The forward primer of each pair was labeled with a fluorescent dye (ROX, FAM, or HEX) during synthesis. PCR products were separated by capillary electrophoresis using the ABI3730xl DNA Analyzer (Applied Biosystems, Carlsbad, CA, USA). Genotypes were determined using Gene-Marker 2.2.0 software (SoftGenetics LLC, State College, PA, USA).

### Phenotypic Data

Ten growth- and wood-property traits were measured in all 700 clones in 2014 in Longshan State Forest Farm, with at least three randomly selected ramets per clone. The growth traits included tree height (H), diameter at breast height (DBH), bark thickness (T), and stem volume (V). The wood-property traits included proportion of heartwood (P), wood basic density (WBD), hygroscopicity (Hy), tracheid length (L), tracheid diameter (D), and the ratio of L to D (L/D). Growth traits, including H, DBH, and T, were measured during field surveys using the method described by Duan *et al*.^50^. V was calculated according to the formula V = 0.000 058 777 042 × D^1.9699831^ × H^0.89646157^. Additionally, a wood core was drilled at breast height from each tree using a tree growth cone and then placed in a plastic tube, which was not completely sealed, to prevent wet rot. P and WBD were measured using the method described by Duan *et al*.^50^. Hy was evaluated using the formula Hy = (W1 −W2)/W2, where W1 and W2 represent the water-saturated weight and oven-dry weight, respectively^47^. L and D were measured using the methods described by Huang *et al*.^51^.

### Data Analysis

Microsoft Excel 2010 and SAS ver. 8.1 (SAS Institute, Cary, NC, USA) were used to examine trait differences in the phenotypic traits; these analyses excluded abnormal data obtained from weak grafts, including the mean value, standard error, amplitude, and coefficient of variation (*CV*). Detailed sampling, measurement methods, phenotypic variations, and phenotypic correlations for these 10 traits were described in a previous study^45^.

Microsatellite data were converted into various formats using Convert 1.3.1^52^ for further analysis. The level of genetic diversity for all loci, including the number of alleles (*N*a), effective number of alleles (*N*e), Shannon index (*I*), observed heterozygosity (*H*o), expected heterozygosity (*H*e), gene flow (*N*m), and F-statistics calculations (*F*_IS_, *F*_IT_, and *F*_ST_), were calculated using GenAlEx 6 software^53^. Polymorphism information content (PIC) was calculated using PowerMarker v. 3.25^54^. Hardy–Weinberg equilibrium (HWE) for all loci was assessed using Arlequin version 3.5^55^ with 100,000,000 steps in the Markov chain and 100,000 dememorization steps^56^. Null alleles were detected using Microchecker 2.2.3^57^. A neutrality test for all loci was performed in Popgene version 1.32^58^. An analysis of molecular variance (AMOVA) was performed to partition the genetic variance among and within the provinces using GenAlEx 6.5^53^ together with Microsoft Excel 2010.

Genetic variation among the samples obtained from six different provinces was evaluated by calculating genetic parameters using GenAlEx 6.5^53^. The *F*_ST_ values were calculated to evaluate the genetic differentiation between any two provinces using FSTAT version 2.9.3^59^. Nei’s genetic distance was estimated by GenAlEx 6.5^53^. The pairwise genetic distance [*F*_ST_/(1 − *F*_ST_)]^60^ (1997) among any two provinces was also estimated.

The population structure of the clones was analyzed using the Bayesian model-based clustering algorithm in STRUCTURE ver. 2.3.1^61^. The software implements the Markov chain Monte Carlo (MCMC) algorithm and a Bayesian framework under admixture model, correlated allele frequencies. Subgroups were identified based on distinctive allele frequencies, and individuals were placed into K clusters by estimated membership probability (Q). In this study, the optimum value of K was determined using the model developed by Evanno *et al*.^62^, with an ad hoc statistic (ΔK), based on the second-order rate of change in the log probability of data between successive K values. The algorithm was run 10 times with a burn-in of 100,000 iterations, followed by 1,000,000 iterations for each value of K and subpopulations (K) ranging from 1 to 20. The height of this modal value was used as an indicator of the strength of the signal that was detected using Structure Harvester^63^. To further compare the evidence for a range of K values, we set Kmin to 1 and Kmax to 20, and the main repeats were 10, using Marverick software with the default parameters^64^. The CLUMPAK web server^65^ was used to visualize the bar plot of the probability of membership from the results of Q-matrix and to evaluate modality.

A core collection was constructed. The optimal sampling method and amount were determined using PowerMarker v. 3.25^54^ based on M and random sampling strategies. The M strategy is also known as the maximum number of alleles strategy^66,67^. The random sampling strategy was the same for all materials, and a certain number of samples was randomly assigned from germplasm materials^68^. The two sampling strategies were compared among 20, 25, 30, 35, 40, 45, 50, 60, 70, 80, 90, 100, 110, 120, 130, 140, 150, 200, 250, 300, and 400 individuals to assess the efficiency of allele sampling using molecular data. For phenotypic data consisting of continuous variables, 100% of the diversity can be sampled based on the precision of classification using PowerCore software^69^. From the two comprehensive sets of results, a core set available for future association mapping was constructed. Simple t-tests were performed for all phenotypic data and for the genetic parameters of the entire collection and the core collection using SAS ver. 8.1 (SAS Institute, Cary, NC, USA).

## Electronic supplementary material

Supplementary information

Table S3

Table S4
