# Supplementary material for: Genetic Characterization of Chinese fir from Six Provinces in Southern China and Construction of a Core Collection
Source: Sci Rep. 2017 Oct 23;7:13814. doi: 10.1038/s41598-017-13219-0 (PMC5653812; doi:10.1038/s41598-017-13219-0)
Supplement: Supplementary file 1 — Supplementary information [file 41598_2017_13219_MOESM1_ESM.pdf]

Genetic Characterization of Chinese fir from Six Provinces in Southern China and Construction of a Core Collection

Hongjing Duan[1], Sen Cao[1], Huiquan Zheng[2], Dehuo Hu[2], Jun Lin[3], Binbin Cui[4], Huazhong Lin[5], Ruiyang Hu[1], Bo Wu[1], Yuhan Sun[1], Yun Li[1*]

[1]Beijing Advanced Innovation Center for Tree Breeding by Molecular Design, National Engineering Laboratory for Tree Breeding；Key Laboratory of Genetics and Breeding in Forest Trees and Ornamental Plants, Ministry of Education; College of Biological Sciences and Technology, Beijing Forestry University, 100083 Beijing, People's Republic of China; E-Mails: duan673356712@126.com (H.D.); sailingcs@163.com (S.C.); hury1102@163.com (R.H.); 6545441823@qq.com (B.W.); syh831008@163.com (Y.S.)

[2]Guangdong Provincial Key Laboratory of Bio-control for the Forest Disease and Pest, Guangdong Academy of Forestry, 510520 Guangzhou, People's Republic of China; zhenghq@sinogaf.cn (H.Z.); hudehuo@163.com (D.H.)

[3]The *ex situ* gene bank of Longshan State Forest Farm, 512221, Guangdong Province, People's Republic of China; m13902346498@163.com.

[4]Department of Biochemistry, Baoding University, 071000, Baoding, Hebei Province, People's Republic of China; cbb0508@163.com.

[5]Fujian Jiangle State-owned Forestry Farm, Fujian 353300, China; E-Mail: fjsmlhz@sina.com.

Figure S1

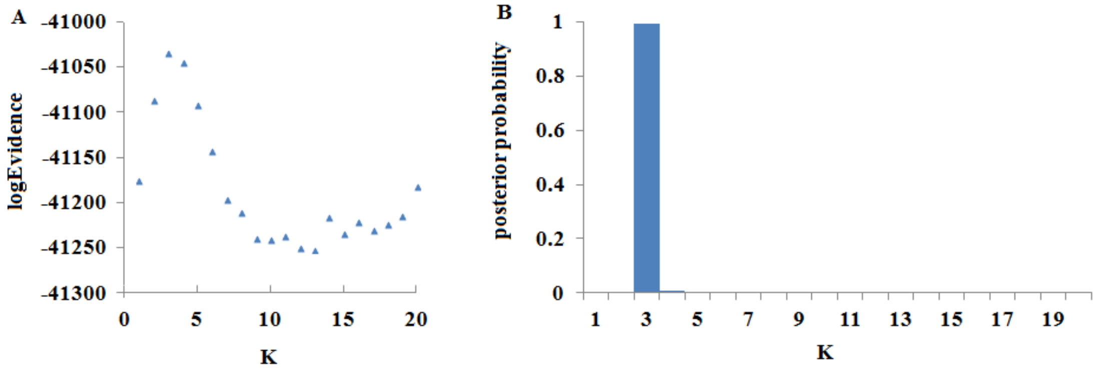

Figure S1. Estimates of the model evidence under the admixture model. **A.** In log space. **B.** In linear space after normalising to sum to 1.

Figure S2

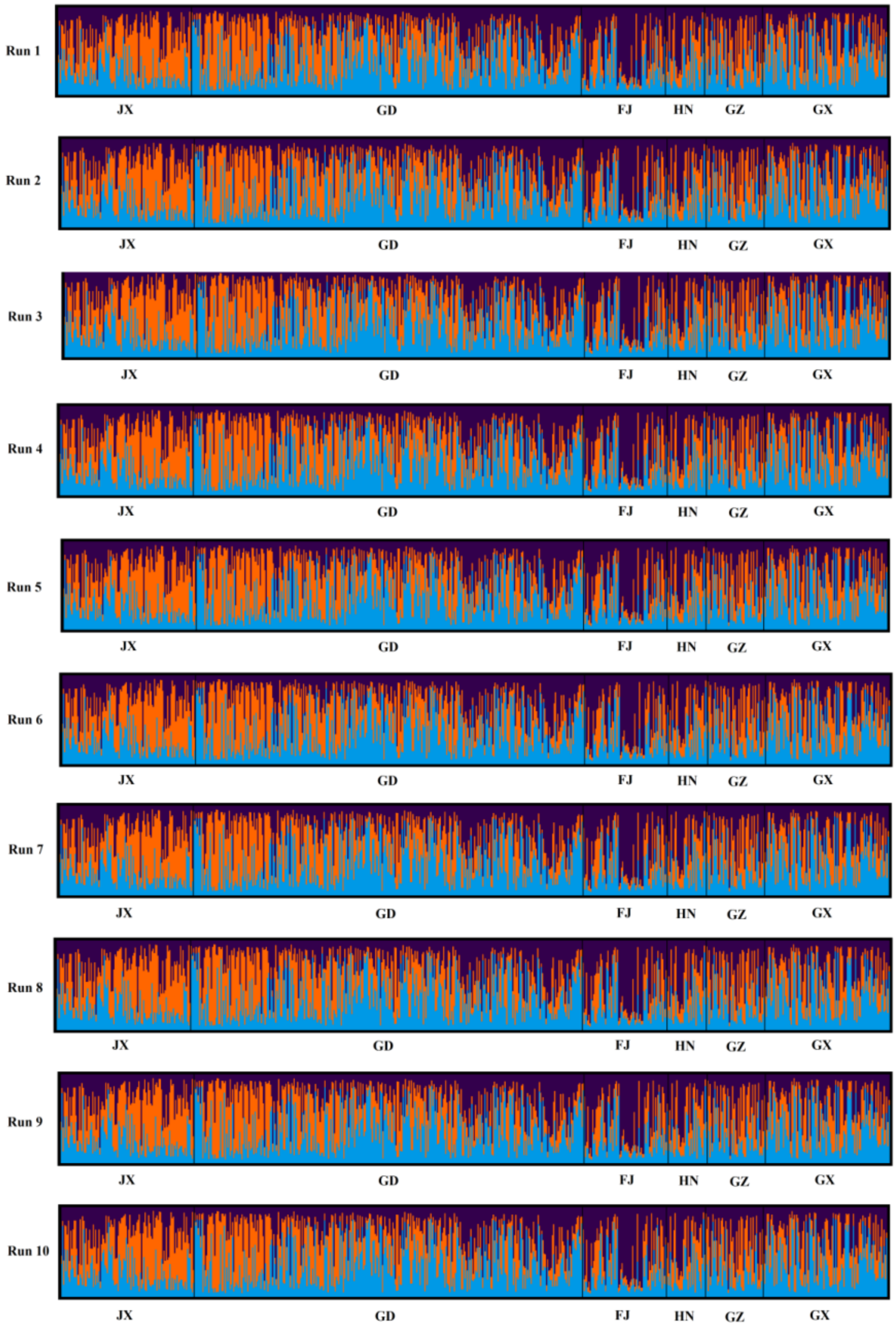

Figure S2. Membership coefficients compared between different runs in STRUCTURE with K = 3.

Figure S3

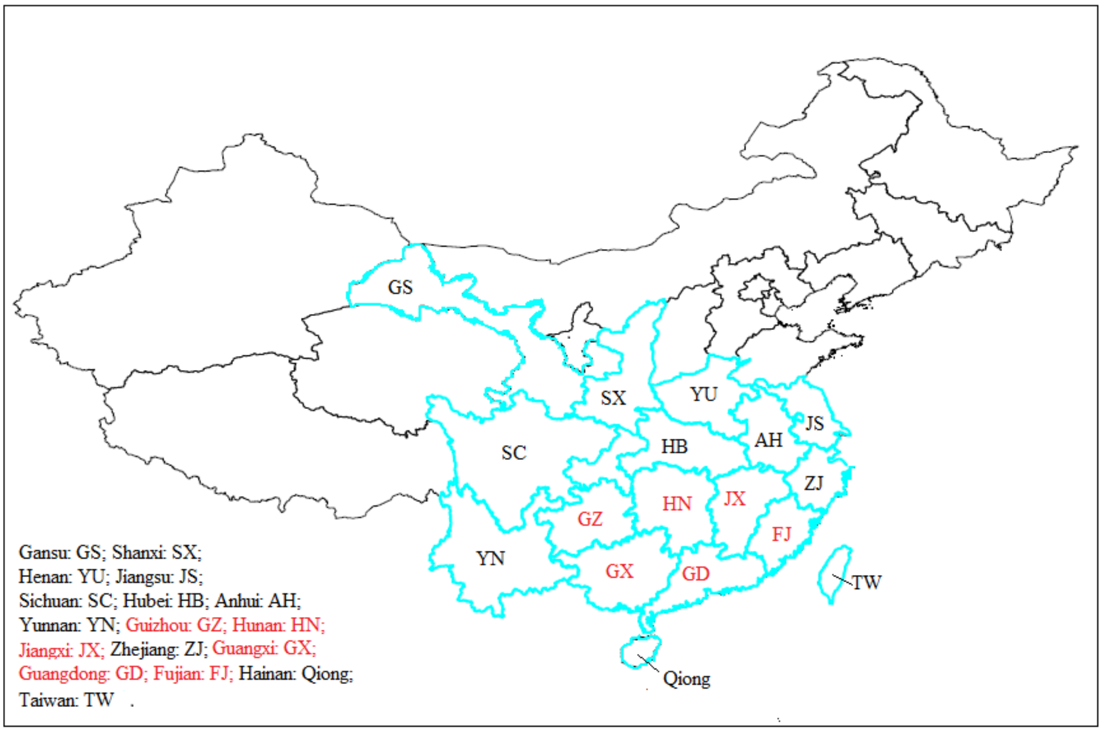

Figure S3. The distribution range of Chinese fir (Red font represents where Chinese fir was collected in this study). The image was generated by the software ArcGIS 10.2 (ESRI, Redlands, CA, USA). http://www.newasp.net/soft/107715.html.

Table S1 Diversity information parameters at 21 SSR loci for Chinese fir.

Locus	*N*a	*N*e	*I*	H_O	*H*e	F_{IS}	F_{IT}	F_{ST}	*N*m	PIC	Null alleles (frequency)
SSR1	18.83	11.05	2.601	0.451	0.908	0.504*	0.514	0.020	12.279	0.92	Yes(0.28)
SSR2	14.17	8.03	2.266	0.809	0.872	0.072*	0.085	0.014	17.969	0.88	Yes(0.06)
SSR3	3.83	1.47	0.588	0.275	0.318	0.136	0.150	0.016	15.368	0.29	No
SSR4	10.33	4.01	1.619	0.735	0.742	0.009	0.026	0.017	14.479	0.72	No
SSR5	7.33	2.53	1.248	0.571	0.596	0.042	0.056	0.015	16.402	0.57	No
SSR6	6.67	2.13	1.067	0.456	0.525	0.130*	0.139	0.010	24.860	0.51	No
SSR7	5.83	3.84	1.456	0.651	0.740	0.119	0.130	0.011	21.608	0.70	No
SSR8	5.17	1.92	0.908	0.463	0.476	0.028	0.035	0.007	35.310	0.45	No
SSR9	8.33	4.33	1.619	0.759	0.769	0.013	0.026	0.013	19.098	0.74	No
SSR10	5.83	2.68	1.145	0.623	0.623	-0.001	0.006	0.007	34.640	0.55	No
SSR11	17.83	10.31	2.507	0.737	0.897	0.178*	0.193	0.018	13.582	0.91	Yes(0.29)
SSR12	6.50	1.54	0.764	0.364	0.342	-0.065	-0.052	0.012	20.929	0.32	No
SSR13	5.67	2.26	0.970	0.577	0.553	-0.042	-0.022	0.020	12.563	0.47	No
SSR14	7.67	2.62	1.168	0.660	0.617	-0.069	-0.063	0.006	42.319	0.52	No
SSR15	5.50	1.96	0.951	0.507	0.487	-0.041	-0.031	0.009	26.678	0.45	No
SSR16	8.50	2.12	1.073	0.559	0.522	-0.071	-0.045	0.024	10.299	0.47	No
SSR17	6.17	1.39	0.639	0.268	0.275	0.023	0.035	0.013	18.984	0.25	No
SSR18	6.17	3.80	1.499	0.771	0.735	-0.049	-0.035	0.013	18.446	0.70	No
SSR19	5.17	1.67	0.751	0.369	0.394	0.062	0.086	0.025	9.871	0.37	No

SSR20	5.83	1.74	0.824	0.411	0.416	0.013	0.032	0.019	12.571	0.36	No
SSR21	13.17	8.40	2.292	0.766	0.880	0.130*	0.145	0.017	14.567	0.89	Yes(0.11)
Mean	8.31	3.80	1.331	0.561	0.604	0.053	0.067	0.015	19.658	0.57	—
C.V	41.41	76.75	45.21	29.41	32.16						
Total	181		—	—							

Na: Number of Different Alleles; *Ne*: Number of Effective Alleles; *I*: Shannon's Information Index; H_O: Observed Heterozygosity; *He*: Expected Heterozygosity; F_{IS}: inbreeding coefficient; F_{IT}: over inbreeding coefficient; F_{ST}: fixation index; *Nm*: Gene Flow; PIC: polymorphic information content; *: significant deviation from Hardy-Weinberg equilibrium (HWE) ($p < 0.05$).

Table S2 The number of genotypes in each cluster.

Substructure	Number of genotypes in the cluster								
	Guangxi	Jiangxi	Hunan	Guizhou	Fujian	Guangdong	Total	Q > 0.8	Q < 0.6
Cluster 1	25	61	8	18	14	111	237	38	112
Cluster 2	44	25	8	12	15	137	241	44	114
Cluster 3	36	27	17	19	42	81	222	26	117

Q: estimated membership probability.

Table S5 Pairwise F_{ST} (lower diagonal) and Nei's standard genetic distances (upper diagonal) among trees from six provinces.

	Guangxi	Jiangxi	Hunan	Guizhou	Fujian	Guangdong
Guangxi	0.000	0.020	0.033	0.023	0.024	0.009
Jiangxi	0.006	0.000	0.039	0.027	0.032	0.009
Hunan	0.011**	0.013**	0.000	0.035	0.049	0.032
Guizhou	0.007**	0.008**	0.012**	0.000	0.040	0.020
Fujian	0.007	0.009**	0.016**	0.012**	0.000	0.021
Guangdong	0.003	0.003	0.011	0.006**	0.006**	0.000

******p < 0.01

Table S6 Geographical parameters of Chinese fir used in this study.

Provinces used in this study	Number of genotypes	Provenances	Longitude (E)	Latitude (N)
Guangxi(GX)	105	Rongshui, Rongan, Liuzhou, Sanjiang, Nandan, Hengxian, Paiyang, Yulin, Ningming, and Hexian	108° 29′ (104°49′–112°08′)	23° 66′ (20°92′–26°40′)
Jiangxi(JX)	113	Quannan, Yifeng, Chongyi, Yudu, Xinfeng, Ji'an, Wuning, Tonggu, and Anfu	116° 26′ (114°05′–118°47′)	26° 64′ (24°13′–29°15′)
Hunan(HN)	33	Jingzhou, Jianghua, and Taoyuan	111° 31′ (108°47′–114°15′)	27° 23′ (30°08′–24°38′)
Guizhou(GZ)	49	Jinping, Liping, Tianzhu, Sandu, Dansai, and Rongjiang	106° 61′ (103°62′–109°59′)	25° 95′ (23°52′–28°37′)
Fujian(FJ)	71	Nanping, Yangkou, Laizhou, Sanming, Datian, Liancheng, and Jianou	117° 95′ (115°50′–120°40′)	23° 66′ (23°33′–28°20′)
Guangdong(GD)	329	Lechang, Liannan, Lianshan, Yangshan, Ruyuan, Renhua, Shixing, Nanxiong, Lianping, Qingyuan, Fogang, Huaiji, Yunan, Xinyi, Meizhou, Raoping, and Jiaoling	113° 54′ (109°75′–117°33′)	22° 86′ (20°20′–25°51′)
